# Editorial: Pharmacological modulation of inflammatory pathways in sepsis: targeting the cytokine storm and septic oxidative stress

**DOI:** 10.3389/fphar.2026.2021242

**Published:** 2026-10-20

**Authors:** Gavin Zhang, Hanna VanBramer, Dawei Wang, Amit Prasad, Qinghe Meng

**Affiliations:** 1 Department of Biology, Tufts University, Medford, MA, United States; 2 Department of Surgery, SUNY Upstate Medical University, Syracuse, NY, United States; 3 Emergency Department, Shandong Provincial Hospital Affiliated to Shandong First Medical University, Jinan, China; 4 Indian Institute of Technology Mandi, Mandi, India

**Keywords:** endothelial dysfunction, immunomodulation, inflammation, mitochondria and oxidative stress, sepsis

## 1 Introduction

Sepsis remains a major clinical challenge despite advances in antimicrobial therapy, source control, hemodynamic resuscitation, and organ support (Singer et al., 2016; Evans et al., 2021). The high mortality associated with sepsis reflects not only overwhelming inflammation but also profound alterations in immune regulation, oxidative balance, mitochondrial function, endothelial integrity, metabolism, and organ homeostasis (Park and Zmijewski, 2017). Historically, pharmacological research has focused heavily on the concept of a systemic “cytokine storm,” prompting extensive investigation of anti-inflammatory therapies. However, the limited success of many approaches targeting individual inflammatory mediators suggests that sepsis cannot be adequately treated by solely suppressing inflammation.

The research topic *“Pharmacological Modulation of Inflammatory Pathways in Sepsis: Targeting the Cytokine Storm and Septic Oxidative Stress”* brought together 21 contributions encompassing experimental studies, mechanistic investigations, natural-product pharmacology, drug repurposing, systematic reviews, network meta-analysis, real-world evidence, and randomized clinical research. Collectively, these studies reveal an important evolution in the field: from targeting individual inflammatory mediators toward restoring immune, mitochondrial, vascular, metabolic, and organ homeostasis.

Here, we highlight several themes emerging from this collection and propose that the next-generation of sepsis pharmacotherapy should prioritize precision, temporally adaptive, host-directed therapy over nonspecific inflammatory suppression.

## 2 Beyond the cytokine storm: inflammation as a network

Inflammation remains central to sepsis pathophysiology. Activation of innate immune receptors induces NF-κB, MAPK, inflammasome, and related pathways, resulting in the production of TNF-α, IL-1β, IL-6, HMGB1, and other mediators. Nevertheless, inflammatory signaling does not occur in isolation; it interacts continuously with oxidative stress, mitochondrial dysfunction, endothelial injury, coagulation, metabolism, and immune-cell dysfunction.

The review of mitochondrial quality control in sepsis highlights this interconnected biology and emphasizes mitochondrial biogenesis, dynamics, and mitophagy as important regulators of cellular homeostasis (Chang M et al.). Likewise, the review of natural products in sepsis-associated organ dysfunction identifies convergent pathways involving NF-κB/NLRP3, Nrf2/HO-1, and AMPK/mTOR, linking inflammation with oxidative stress, metabolism, mitochondrial biology, and cell death (Wang J et al.).

These findings support a broader concept: sepsis is a network disease, not a single-pathway inflammatory disorder. Consequently, reducing one cytokine may be insufficient if mitochondrial injury, endothelial dysfunction, or immune dysregulation continues to drive organ injury.

## 3 Mitochondria and oxidative stress: therapeutic targets rather than bystanders

Mitochondrial dysfunction serves as a vital link between inflammation and cellular injury. During sepsis, impaired oxidative phosphorylation, excessive reactive oxygen species generation, ATP depletion, and mitochondrial damage can promote further inflammatory signaling and cellular dysfunction. This creates a potentially self-amplifying cycle: inflammation → oxidative stress → mitochondrial dysfunction → cellular injury → DAMP release → further inflammation. The mitochondrial quality-control review suggests that modulating mitochondrial biogenesis, dynamics, and mitophagy may offer therapeutic opportunities (Chang M et al.). Experimental evidence from Xiangdan injection further supports this concept, indicating attenuation of inflammatory responses and cardiac injury, as well as mitochondrial protection, in experimental sepsis (Xu J et al.).

However, significant translational limitations remain. Many preclinical studies define “mitochondrial protection” using morphology or selected protein markers rather than direct measurements of mitochondrial function. Future studies should therefore incorporate mitochondrial respiration, ATP generation, membrane potential, mitophagy flux, and mitochondrial biogenesis when feasible. The critical question is not simply whether a treatment reduces oxidative stress, but whether it restores cellular bioenergetic function.

## 4 NETs, endothelial dysfunction, and microcirculatory failure

The collection also emphasizes the important role of vascular dimension of sepsis. NETs provide a key mechanistic connection between innate immune activation and vascular injury. Excessive NET formation can contribute to endothelial damage, coagulation activation, vascular permeability, immunothrombosis, and microcirculatory dysfunction (Zhang P et al.). This perspective is important because cytokines represent only part of the pathway leading to organ failure. Ultimately, tissue injury is influenced by endothelial integrity, perfusion, oxygen delivery, and microvascular blood flow.

The DHA study provides complementary evidence. DHA reduced inflammatory responses, neutrophil adhesion, and microcirculatory abnormalities in experimental sepsis (de Moraes BPT et al.). An increased formation of pro-resolving mediators was also reported, but this was determined using enzyme immunoassays, which have limited selectivity in complex biological samples (Naicker et al., 2026; Schebb et al., 2026). These findings therefore require verification by LC-MS/MS. These findings suggest that targeting inflammatory cell–vascular interactions may provide an alternative or complementary strategy to direct cytokine inhibition.

The emerging NET-endothelium-microcirculation axis therefore deserves greater attention. Future studies should determine whether NET-directed therapies can improve organ perfusion and clinical outcomes without compromising antimicrobial host defense (Zhang P et al.).

## 5 Immunomodulation: Suppression or restoration?

Perhaps the greatest challenge to the cytokine-storm paradigm is the recognition that hyperinflammation and immunosuppression may coexist during sepsis. Patients may transition from excessive inflammatory activation to impaired immune competence or exhibit features of both states simultaneously.

The systematic review and network meta-analysis in this Research Topic synthesized 76 randomized trials involving 22,194 patients and evaluated multiple immunomodulatory interventions (Luo R et al.). Several therapies, including ulinastatin, ulinastatin plus thymosin-α1, and polyunsaturated fatty acids, showed potential benefits, although heterogeneity and limitations in the certainty of the evidence remain.

These findings suggest that the key question is no longer whether inflammation should be suppressed, but rather: Which biological phenotype should receive which intervention, and when? This distinction may be critical for successful clinical translation. A patient with dominant hyperinflammation may require targeted anti-inflammatory therapy, whereas another with immune paralysis may require immune restoration. Treating these biologically distinct states as a single syndrome is unlikely to produce consistent benefits.

## 6 Metabolic and vascular pharmacology: expanding the therapeutic toolbox

Several contributions demonstrate that pharmacological modulation of sepsis may extend beyond conventional immunology.

In a randomized controlled study of patients with septic shock, anisodamine hydrobromide was associated with reduced 28-day mortality, with lactate-related variables contributing to the observed treatment effect (Yang X et al.). These findings present the possibility that improving microcirculation and tissue perfusion may be therapeutically relevant in addition to suppressing inflammation. Liraglutide provides another example. The study examining GLP-1 receptor agonism during sepsis demonstrated effects on retinal endothelial function and oxidative stress, suggesting that metabolic signaling may influence vascular injury during systemic inflammation (Bohm EW et al.). Finerenone further illustrates this concept. In a real-world cohort of patients with diabetic kidney disease, finerenone use was associated with lower risks of sepsis-induced cardiomyopathy and mortality compared with steroidal mineralocorticoid receptor antagonists (Wu JY et al.). Although observational data cannot establish causality, these findings provide a rationale for prospective investigation.

Together, these studies suggest that metabolic and cardiovascular drugs may possess host-directed effects that extend beyond their traditional indications.

## 7 Natural products and drug repurposing: promise with important caveats

Natural products and traditional Chinese medicines constitute a substantial component of the Research Topic, including studies of artesunate, Dachengqi Decoction, Xiangdan injection, sophoridine, ganoderic acid A, Handelin, DHA, and related agents (Jena SP et al., Wang J et al., Xu J et al., Ma H et al., Masic T et al., Chen D et al., Pan GT et al., Hu H et al., Xie W et al., Hong C et al., de Moraes SPT et al., Itoh K et al.).

Their potential advantage is multitarget activity. Because sepsis involves interconnected pathological processes, simultaneous modulation of inflammatory, oxidative, mitochondrial, metabolic, and cell-death pathways may be advantageous. However, multitarget activity is also a translational challenge. Variable composition, uncertain bioavailability, poorly defined pharmacokinetics, difficulty establishing clinically achievable concentrations, and limited randomized clinical evidence remain major obstacles (Wang J et al.). The ganoderic acid A study illustrates an encouraging direction by integrating multi-omics approaches with experimental validation and identifying TNF-α as a candidate target in sepsis-related liver injury (Hu H et al.). Similarly, multi-omic profiling of Handelin identified proteasome subunits PSMA7 and PSMB2 as potential targets, suggesting that proteostasis may represent an underexplored therapeutic dimension of sepsis (Chen D et al.).

The Bergenia ciliata study demonstrates how metabolomics, ADME analysis, network pharmacology, molecular docking, molecular dynamics, cellular experiments, and animal studies can be integrated to investigate drug-induced nephrotoxicity (Jena SP et al.). Importantly, however, this model concerns artesunate-induced nephrotoxicity rather than sepsis itself, illustrating the need to distinguish mechanistic relevance from direct evidence of sepsis efficacy. Artesunate is more directly relevant to sepsis. A systematic review and meta-analysis of animal studies reported beneficial effects of artesunate on survival and inflammatory or organ-injury outcomes, although heterogeneity and limitations of preclinical evidence remain (Xie W et al.).

Thus, future natural-product research should progress from pathway association toward validated molecular targets, pharmacokinetic exposure, target engagement, functional efficacy, and clinical translation.

## 8 Organ-specific pharmacology and precision dosing

The Research Topic also highlights that systemic inflammation ultimately produces tissue-specific injury. Kidney, heart, brain, liver, and vascular injury may require distinct therapeutic strategies.

The vancomycin study provides an important example of precision pharmacology. In septic patients, higher vancomycin concentrations were associated with increased AKI risk, and hyperlactatemia modified this relationship (Tang J et al.). This emphasizes that drug toxicity depends not only on exposure but also on the physiological context of the patient. Similarly, a systematic review comparing vasopressin and norepinephrine found potential renal benefits associated with vasopressin, including lower serum creatinine and reduced renal replacement therapy. However, the effects on AKI were less consistent (Wang H et al.).

These findings reinforce an important principle: sepsis pharmacology is not only about discovering new drugs but also about optimizing existing therapies according to patient physiology, organ function, and drug exposure.

## 9 What remains unresolved?

The collection also highlights several important gaps. First, biological heterogeneity remains insufficiently incorporated into clinical trials. Biomarkers reflecting inflammation, immune competence, endothelial injury, mitochondrial dysfunction, and metabolism may help identify patients most likely to respond to specific interventions. Second, timing matters; a treatment beneficial during early hyperinflammation may be ineffective, or potentially harmful, during subsequent immunosuppression. Third, mechanistic evidence requires stronger causal validation. Molecular docking, transcriptomic associations, and pathway enrichment are valuable for hypothesis generation but should be supported by target engagement, genetic manipulation, rescue experiments, and functional assays. Fourth, preclinical models need greater clinical relevance. Age, sex, diabetes, obesity, chronic kidney disease, immunosuppression, infection characteristics, antibiotic therapy, fluid resuscitation, and vasopressor treatment should be incorporated where appropriate. Finally, combination therapy should be mechanism-guided rather than empiric. The objective should not simply be to combine drugs with different mechanisms, but to target complementary biological abnormalities in appropriately selected patients (Itoh K et al.).

## 10 Conclusions and future perspective

The 21 contributions in this Research Topic collectively demonstrate that sepsis pharmacology is moving beyond a cytokine-centered paradigm toward a more integrated understanding of inflammatory, oxidative, mitochondrial, vascular, metabolic, and immune dysfunction. The cytokine storm remains an important component of sepsis, but it represents only one element of a complex pathological network. Inflammation interacts with oxidative stress; oxidative stress damages mitochondria; mitochondrial dysfunction amplifies inflammatory signaling; activated neutrophils generate NETs; NETs contribute to endothelial injury and microvascular dysfunction; and tissue hypoperfusion further aggravates metabolic and mitochondrial failure (Chang M et al., Zhang P et al., Wang J et al., de Moraes BPT et al.).

The therapeutic challenge therefore to interrupt these self-amplifying processes while preserving antimicrobial defense. The next-generation of sepsis pharmacotherapy should therefore shift from suppressing inflammation to restoring homeostasis. Achieving this goal will require integration of immune phenotyping, mitochondrial biology, endothelial and microvascular assessment, metabolic profiling, pharmacokinetics, and organ-specific biomarkers. Ultimately, the goal should not be to discover a universally effective anti-inflammatory drug. Rather, sepsis treatment should evolve toward the right intervention for the right biological phenotype at the right time. This transition, from the generalized concept of a cytokine storm to precision host-directed therapy, may represent one of the most promising directions for improving sepsis outcomes.
